# Social withdrawal behaviour in Nepalese infants and the relationship with future neurodevelopment; a longitudinal cohort study

**DOI:** 10.1186/s12887-024-04658-6

**Published:** 2024-03-18

**Authors:** Ingrid Kvestad, Manjeswori Ulak, Suman Ranjitkar, Merina Shrestha, Ram K. Chandyo, Antoine Guedeney, Hanne C. Braarud, Mari Hysing, Tor A. Strand

**Affiliations:** 1 Regional Centre for Child and Youth Mental Health and Child Welfare, NORCE Norwegian Research Centre, Bergen, Norway; 2 Department of Research, Innlandet Hospital Trust, Lillehammer, Norway; 3 Centre for International Health, University of Bergen, Bergen, Norway; 4 Department of Pediatrics, Institute of Medicine, Child Health Research Project, Tribhuvan University, Kathmandu, Nepal; 5 Department of Community Medicine, Kathmandu Medical College, Kathmandu, Nepal; 6 University Paris Cité, Paris, France; 7 Department of Psychosocial Science, Faculty of Psychology, University of Bergen, Bergen, Norway

**Keywords:** Early child development, Infant social withdrawal, Nepalese infants

## Abstract

**Background:**

Social withdrawal in infants may be a signal of distress and a precursor for non-optimal development.

**Objective:**

To examine the relationship between infant social withdrawal and neurodevelopment up to 4 years in Nepalese children.

**Methods:**

A total of 597 Nepalese infants 6–11 months old were assessed with the modified Alarm Distress Baby Scale (m-ADBB), and of these, 527 with the Bayley Scales of Infant and Toddler Development 3rd edition (Bayley-III) during early childhood, and the Wechsler Preschool and Primary Scale of Intelligence (WPPSI-IV) and NEPSY-II subtests at 4 years. We examined whether social withdrawal defined by the m-ADBB was associated with neurodevelopmental scores in regression models.

**Results:**

Children socially withdrawn in infancy had lower Bayley-III language scores (-2.6 (95% CI -4.5, -0.7)) in early childhood. This association seems to be driven by the expressive communication subscale (-0.7 (95% CI -1.0, -0.3)), but not the receptive communication subscale (-0.2 (95% CI -0.6, 0.1)). There were no differences in the other Bayley-III scores or the WPPSI-IV and NEPSY-II scores at 4 years in children who were socially withdrawn or not.

**Conclusion:**

Social withdrawal in infancy was reflected in early language development but not cognitive functioning at 4 years.

**Supplementary Information:**

The online version contains supplementary material available at 10.1186/s12887-024-04658-6.

## Introduction

Typically, infants develop the skill to relate to the social world through close interactions with significant caregivers during the first 2 months after birth [1]. Although infants differ individually in how they relate to others for instance due to differences in temperament, all typically developing infants are responsive in the interaction with an adult. Withdrawal behaviour in the infant is a normal feature and a way to regulate social interactions with others. Sustained social withdrawal in infants, however, is considered an early warning sign of infant distress and a risk factor for non-optimal development [1].

Sustained social withdrawal behaviour may be a result of both biological determinants within the infant and qualities in the social environment. From a biological perspective, adequate health and nutrition are prerequisites for the infant’s ability to engage with and benefit from its surroundings [2]. Prolonged infections, chronic diseases, nutrient deficiencies and undernutrition are examples of conditions early in life that may have consequences for the infant’s ability to elicit social interactions from the surroundings, and which may cause sustained withdrawn behaviour [3, 4]. Moreover, infants born premature [5] or with low birth weight [6] have shown more social withdrawal behaviour than infants born to term or with normal birth weight. As a sign of dysregulation in the parent-infant synchrony, infant social withdrawal could also be a result of disturbances in the social environment such as caregiver distress [6]. There is an extensive literature linking maternal mental health and emotional distress to social withdrawal behaviour in infants [5, 7, 8], demonstrating the essential part played by caregivers in infant social development.

The Alarm Distress Baby Scale (ADBB) is a screening tool for social withdrawal in infants and young children 2 to 24 months old that can be scored by clinicians in routine clinical assessments [3]. The usability of the scale has been demonstrated in many studies across several countries [1] and a modified version of the scale (the m-ADBB) with a simpler mode of scoring and fewer scoring categories has also been developed for clinicians and researchers to reach reliable measures more easily [9]. The full ADBB scale examines social withdrawal through eight domains: Facial expression; Eye contact; General level of activity; Self–stimulating gestures; Vocalizations; Briskness of response to stimulation; Relationship with the observer, and the Capacity to attract and maintain attention with the observer [1]. The modified ADBB (m-ADBB) examines social withdrawal through only five domains: Facial expression; Eye contact; Vocalization; Activity level, and Relationship with the observer, and has a simplified mode of scoring [9]. This modified version has been used both in high-income [10–12] and low-income countries [13, 14].

Few studies have investigated the relation between early social withdrawal behaviour and future neurodevelopment and mental health. In a longitudinal study, the ADBB score measured at approximately 6 months was associated with the Cognitive and Language scales of the Bayley Scales of Infant and Toddlers Development, 3rd edition (Bayley-III), as well as the Social and Communication scales of the Behaviour Assessment System for Children when the children were 30 months of age [15]. The French mother–child cohort study, the EDEN study, suggest that children classified as social withdrawn at 12 months had lower scores on the Wechsler Preschool and Primary Scale of Intelligence 3^rd^edition (WPPSI-III) at 5–6 years compared to children who were not socially withdrawn [16]. Moreover, scores on the ADBB scale measured at 12 months were associated with the conduct problem subscale of the Strengths and Difficulties Questionnaire (SDQ) at 3 years, and with SDQ peer problems and prosocial subscale and total difficulties scores at 5 years [17].

Taking previous studies into account, tracking social withdrawal in infancy could be a critical measure to identify infants at risk of adverse development. However, while studies mentioned above are mainly from French study populations, less is known about the relation between early sustained social withdrawal in infants and later neurodevelopment in low-to-middle-income populations. In these low-resource contexts, vulnerable infants encounter a range of risk factors for adverse development [18], such as infections, nutrient deficiencies, and impaired growth [19–21]. Moreover, as a consequence of poverty and poor maternal health, prematurity and low birth weight are prevalent in these low-resource settings [22]. Infants and young children also face a risk of lack of stimulation and learning opportunities [23, 24].

In a large community-based study in Bhaktapur, Nepal, we have previously demonstrated that social withdrawal was common among 597 Nepalese infants 6 to 11 months with approximately 11% having m-ADBB scores above the cut off for social withdrawal [14]. The primary objective of the current study was to investigate the relationship between early infant social withdrawal behaviour and neurodevelopment through examining the associations between the m-ADBB score in infancy and the neurodevelopmental scores at four time points during early childhood.

## Methods

### Study design

The infant participants were part of a community-based, double-blind, randomized controlled trial on the effect of 2–3 recommended daily allowances (2 μg) of vitamin B12 on neurodevelopment, growth, and haemoglobin concentration [25, 26]. Main results from the study showed no effect of B12 supplementation on any of the clinical or functional outcomes, despite an excellent metabolic response [26]. The study was conducted in Bhaktapur municipality and the surrounding peri-urban communities located 15 km east of the capital city Kathmandu. The study has ethical clearance from the Nepal Health Research Council (NHRC, #233/2014) and the Regional Committee for Medical and Health Research Ethics (REC # 2014/1528) in Norway. Written informed consent from the parent and/or legal guardian for study participation was obtained after providing thorough information on study procedures.

### Procedure

Eligible children were identified by field workers from immunization clinics or by door-to-door home visits. We included 600 children 6 to 11 months with a length for age < -1 z-score, who planned to reside in Bhaktapur municipality and the surrounding areas for the next 12 months and where caregivers were available for informed consent. Exclusion criteria included lack of consent and taking or planning to take supplements that contained vitamin B12. Children were also excluded if they had severe systemic illness requiring hospitalization, if they were severely malnourished (weight for length < -3 z-scores), severely anaemic (Hb < 7 g/dL) or had ongoing acute infections or disease. In case of acute infections, children received treatment and were screened again for eligibility after recovery [25, 26]. Enrolment procedures included anthropometric measurements, collection of child and family demographics, and neurodevelopmental assessment. Following enrolment, the family was visited by trained field workers to measure the home environment. The study children were subsequently invited every 12 months to the study clinic for follow up assessments of neurodevelopment and socio-emotional development until they were approximately 4 years old [27]. From the 600 enrolled children, we have developmental and cognitive scores at 4 timepoints in 527 children up to 4 years of age.

### Infant social behaviour

We used the m-ADBB to measure social withdrawal in the infants when they were 6–11 months [9]. The m-ADBB consists of five items that are scored by an examiner while interacting with the infants. These items are Facial expression; Eye contact; Vocalization; General level of Activity, and Relationship with the examiner. The items are scored on a three-point scale: Satisfactory (0), Possible Problem (1) and Definite Problem (2) with thoroughly described scoring criteria. Scoring is not adjusted for prematurity. The total score ranges from 0–9, with higher scores suggesting more social withdrawal behaviour. Scores above 2 have been suggested to denote social withdrawal [9].

Standardization and quality control procedures for the current study have been thoroughly described elsewhere [14]. Videos from the enrolment procedures of the infants interacting with a study physician or supervisor were scored by one of three certified ADBB scorers (SR, MU and MS), with 7.5% double scored by a fourth scorer (IK) to measure interrater agreement reaching an intraclass correlation (ICC) of 0.85 for the total score [14]. In the standardization process, some additional clarifications to the instructions were made to secure reliable scoring.

### Child and family characteristics

During the enrolment procedures we measured weight and length, collected blood and asked caregivers about child characteristics such as birth weight (low birth weight defined as < 2500 g), gestational age (preterm birth defined as < 37 weeks) and child history of hospitalization the first month of life (yes/no); parental characteristics such as age, literacy and occupation; household characteristics such as living in nuclear family or not, house ownership (yes/no) and number of rooms in the home. Weight was measured with a portable electronic scale that measures to the nearest 0.01 kg, and length (Seca) was measured according to standard guidelines at the study clinic. The anthropometric measures were converted to z-scores based on WHO standards [28].

### Neurodevelopment

The neurodevelopmental assessments for the study were performed by trained psychologists in designated well-lit and ventilated rooms free from distraction at the study clinic [29]. Ahead of starting the study assessments, standardisation exercises were performed for 20 children per tester for each test. Double scoring was done throughout the study in 10% of all assessments by two psychologists. We attained an ICC of > 98% for both standardization and double scoring, indicating excellent inter-rater agreement.

At 6–11 months, 18–23 months, and 30–35 months we used the Bayley Scale of Infant and Toddler development 3rd ed. (Bayley-III) to measure early child development. The Bayley-III is a comprehensive assessment tool of developmental functioning in infants and toddlers aged 1–42 months consisting of a cognitive, language (receptive and expressive communication), motor (fine and gross motor) and a socio-emotional scale [30]. For the current analyses, we used the cognitive, language, motor, and socio-emotional composite scores [expected mean (SD) 100 (15)] and receptive and expressive communication subscales [expected mean (SD) 10 (3)]. Raw scores were converted to composite and subscale scores based on US norms [31].

At 42–47 months (approximately 4 years), we measured cognitive abilities with the Wechsler Preschool and Primary Scale of Intelligence, 4th edition (WPPSI-IV) and three NEPSY-II subtests. WPPSI-IV is a clinical assessment tool of intellectual ability in children [32]. We administered 5 subtests: Information, Receptive vocabulary, Block Design, Picture Memory and Zoo Locations [expected mean (SD) 10 (3)] summing up to one full scale IQ score (FSIQ) and three index scores: The Verbal comprehension index (VCI), the Visuo spatial index (VSI) and the Working memory index (WMI) [expected mean (SD) 100 (15)]. The NEPSY-II is a neuropsychological cognitive assessment tool consisting of 32 subtests in six functional domains for children aged 3–16 years [33]. The NEPSY-II is flexible, and the selection of subtests can be tailored for the specific assessment. In the current study, we administered the following three age-appropriate subtests: Affect Recognition, Statue and Geometric Puzzles. The NEPSY-II raw scores are converted to scaled scores [expected mean (SD) 10 (3)] based on US norms, with a total possible range from 1 to 19 [34].

### Statistical analyses

Demographic and clinical characteristics of the sample are presented using numbers (N) and percentages (%), and by means and standard deviations (SD). Associations between the m-ADBB scores and the Bayley-III composite scores at 6–11 months, 18–23 months and 30–35 months were estimated using linear mixed models. The m-ADBB scores were used dichotomized on non-withdrawn (*n* = 466) vs. withdrawn infants (*n* = 61) (cut off ≥ 2). The m-ADBB score, and time (6–11 months, 18–23 months, and 30–35 months) were included as fixed effects, and the individual as a random effect. The analyses were adjusted for determinants for the m-ADBB score identified from a selection of available candidate variables (Supplementary Tables 1 and 2). In the linear mixed models, we also included an interaction term between time and the ADBB variable. Based on the results from the predefined analyses, post hoc we repeated the linear mixed models using the receptive and expressive communication subscale scores as outcomes to further examine potential mechanisms between the social withdrawal behaviour and language development in early childhood. Marginal means from the mixed models are depicted graphically.

In the same longitudinal sample, we also present the mean cognitive scores (WPPSI-IV FSIQ, VCI, VSI and WMI and the NEPSY-II affect recognition, geometric puzzles, and statue) at 42–47 months in infants categorized as socially withdrawn and not at age 6–11 months. In regression models, we calculated the mean difference (95% CI) of WPPSI and NEPSY scores and present both crude and adjusted estimates. Post hoc we repeated the regression analysis using the WPPSI-IV subtests as outcomes to further explore potential mechanisms between social withdrawal behaviour and cognition in early childhood.

Although results from the main trial showed no effect of the intervention (i.e. vitamin B12 supplementation) we repeated all regression analyses, including the intervention group in the models. All analyses were carried out in Stata version 18 (StataCorp, College Station, Texas, USA).

## Results

Demographic characteristics of the 527 infants 6–11 months with ADBB measurements are presented in Table 1. Mean age of the children at enrolment was 8 (1.8) months and 51.2% were male. In the sample, approximately 20% had a birth weight of less than 2500 g, 11% were born preterm and 33% were stunted (length for age z-score < -2). Of the mothers, 35% reported to have an educational level up to grade 5 or were illiterate, and 45% of the families stayed in rented homes.

**Table 1:** Baseline Characteristics in a longitudinal sample of 527 Nepalese infants 6–11 months old

**Child characteristics:**	
Mean Age of child (months), mean (SD)	8 (1.8)
Male child, n (%)	270 (51.2%)
Birth weight (grams), mean (SD)	2781.8 (500.0)
Preterm birth, n (%)	57 (10.8%)
Low birth weight (< 2500 gm), n (%)	105 (19.9%)
Hospitalized prior to enrolment, n (%)	52 (9.9%)
**Nutritional status of infants:**	
Underweight (weight for age Z score ≤ 2), n (%)	104 (19.7%)
Stunting (length for age Z score ≤ 2), n (%)	174 (33.1%)
Anaemia (haemoglobin < 110 g/L), n (%)	337 (63.9%)
**Demographic features:**	
Mother’s age, mean (SD)	27.7 (4.6)
Literacy of mother, n (%):	
a. Illiterate or up to grade 5	186 (35.2%)
b. Grade 5 to 10	102 (19.4%)
c. SLC to grade 12	134 (25.4%)
d. Bachelor	82 (15.6%)
e. Master or above	23 (4.4%)
Literacy of father^*^, n (%):	
a. Illiterate or up to grade 5	184 (34.9%)
b. Grade 5 to 10	116 (22.0%)
c. SLC to grade 12	130 (24.7%)
d. Bachelor	65 (12.3%)
e. Master or above	31 (5.9%)
Family staying in joint family, n (%)	269 (51.0%)
Family residing in rented house, n (%)	237 (45.0%)
Kitchen and bedroom in the same room, n (%)	247 (46.9%)

Values are N (%) unless otherwise specified, ^*^missing information in one participant

**Fig. 1:**
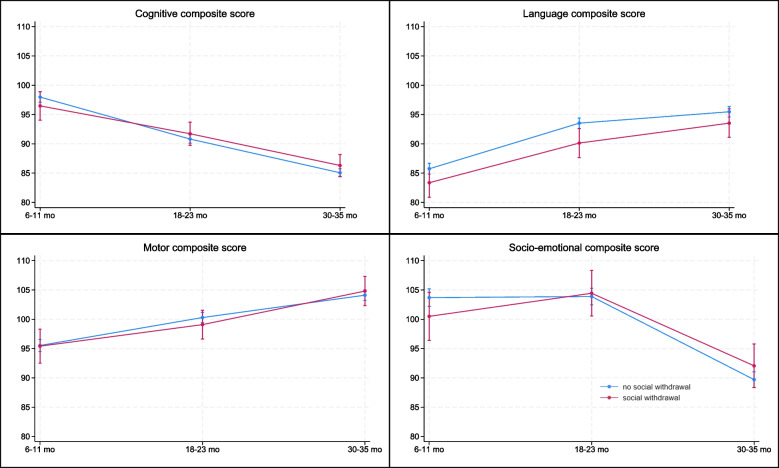
Bayley Scales of Infant and Toddler Development, 3rd edition composite scores in early childhood in Nepalese children socially withdrawn (*n* = 61) or not (*n* = 466) in infancy. Legend: Marginal means from linear mixed effects models with the dichotomized m-ADBB score and time included as fixed effects and the individual as random effect and adjusted for age at ADBB measurement, whether family lives in a rented house, low birth weight and whether child is cared for by one of three regular carers when parents are away. In these models we also included an interaction term between time and the ADBB variable. Blue line represents children with no social withdrawal behaviour in infancy, red line children with infant withdrawal in infancy

Figure 1 graphically presents the marginal means of the Bayley composite scores at 6–11, 18–23, and 30–35 months by infants categorized as socially withdrawn and not when they were 6–11 months. None of the interaction terms in the linear mixed models were significant. In linear mixed models without the interaction term included, being socially withdrawn in infancy was associated with lower Bayley-III language (-2.6 (95% CI -4.5, -0.7)) composite scores, but not with cognitive (0.6 (95% CI-0.8, 1.0)), motor (-0.1 (95% CI -2.1, 1.9)) or socio-emotional (0.3 (95% CI-2.5, 3.2)) composite scores (data not shown).

Post hoc, to further explore associations between early social withdrawal behaviour and language development between 6 and 35 months, we repeated the linear mixed models with the receptive and expressive communication subscales as outcomes (Supplementary Fig. 1). In these models, the expressive communication subscale was associated with early social withdrawal in infancy (-0.7 (95% CI -1.0, -0.3)), while the receptive communication subscale was not (-0.2 (95% CI -0.6, 0.1)).

Table 2 shows the mean (SD) WPPSI-IV scores by children socially withdrawn in infancy or not, and the mean difference of the scores between the groups in crude and adjusted models. There are no differences in cognitive scores at 4 years between children socially withdrawn and not in infancy. In the interest to further explore the associations between social withdrawn behaviour and language development found in early childhood, we also did these regression analyses with each WPPSI-IV subtest score as an outcome (Supplementary Table 3). These post-hoc analyses confirmed the main analysis of no association with cognition at 4 years of age. Including intervention group in the regression models did not alter the estimates or confidence intervals.

**Table 2:** Wechsler Preschool and Primary Scale of Intelligence, 4th edition total and index scores and NEPSY-II subtest scores in 527 children 4 years of age by being socially withdrawn or not in infancy

**WPPSI-IV, 42–47 months**	**Infants with social withdrawal behaviour *n* = 61**	**Infants without social withdrawal behaviour *n* = 466**	**mean diff (95%CI)**^**a**^	**mean diff (95%CI)**^**b**^
			**crude**	**Adjusted**^**a**^
Full scale	84.3 (8.3)	84.8 (8.6)	-0.5 (-2.8, 1.8)	0.5 (-2.2, 2.3)
Verbal comprehension index	82.5 (6.5)	84.2 (8.0)	-1.7 (-3.8, 0.4)	-1.4 (-3.5, 0.7)
Visuo-spatial index	86.6 (8.4)	85.5 (8.4)	1.2 (-1.1, 3.4)	1.6 (-0.7, 3.9)
Working-memory index	102.0 (14.6)	103.8 (13.1)	-1.8 (-5.4, 1.7)	-0.9 (-4.4, 2.7)
NEPSY-II, 42–47 months				
Affect recognition	8.2 (2.0)	8.1 (2.0)	0.1 (-0.5, 0.6)	0.3 (-0.3, 0.8)
Geometric puzzles	9.1 (2.9)	9.1 (2.8)	0.01 (-0.7, 0.8)	0.3 (-0.5, 1.0)
Statue	9.8 (3.3)	10.3 (3.3)	-0.4 (-1.1, 0.4)	-0.3 (-1.2, 0.6)

^a^generalized linear models

^b^adjusted for age at ADBB measurement, whether family lives in a rented house, low birth weight and whether child is cared for by one of three regular carers when parents are away

## Discussion

In the current study, we aimed to examine the relationship between social withdrawal behaviour in infancy and neurodevelopment measured within the first four years of life. Social withdrawal behaviour were associated with poorer language development in early childhood, but not with cognitive, motor, or socio-emotional development. There were no differences in cognitive function at 4 years in the group of children categorized as socially withdrawn or not in infancy.

This is the first study to examine infant social withdrawal behaviour in a South Asian setting and the association with early child development and cognition the first years of life. Our findings suggest that being socially withdrawn in infancy, measured by the m-ADBB, is associated with language development up to the child`s 3rd birthday. The language subscales of the Bayley-III, in particular early in development, are related to social-communicative skills and how the infant relates to others [30]. A previous study found an association between early social withdrawal behaviour and language and social communicative skills at 30 months, which is in accordance with the current findings [15]. Further exploring the associations between early social withdrawal in infancy and language subscales, we find that the association is driven by the expressive communication subscale and not the receptive communication subscale. In other words, children evaluated to be socially withdrawn in infancy had poorer expressive communication skills in early childhood. It should be noted that in general, this sample had high scores on the vocalization items of the m-ADBB, suggesting low levels of vocal utterances among the children when they were infants [14]. Low levels of vocal utterances are also reflected in the Bayley-III expressive communication subscale in the same study sample [29]. Our results could suggest that higher m-ADBB is an early risk indicator for later expressive language problems. However, the differences between groups were small (2.6 language composite scores (i.e. development quotient) and 0.7 expressive subscale score), and the clinical significance may be limited as underscored by the fact that the association between early social withdrawal and language development at 4 years is negligible. Moreover, it is important to underscore that the findings on the difference between expressive and receptive subscale scores are based on post hoc analysis included after completion of the predefined analysis. Therefore, findings should be interpreted with care and new studies are warranted to further understand the associations and mechanisms involved between early social withdrawal and language development.

Infant social withdrawal was not associated with the other development domains throughout early childhood, or with intellectual functioning when the children were approximately four years. The lack of associations with the WPPSI-IV and NEPSY-II scores at 4 years contrasts with previous studies. In for instance the EDEN study, children socially withdrawn at 12 months had lower intelligence scores on the WPPSI-III and more mental health problems at 5–6 years [16, 17]. This lack of associations could have several explanations. The psychometric properties of the m-ADBB in this setting have been questioned and there is a risk for type II errors and that we are not able to identify relationships that are true in the population. It could also be that the consequences of early social withdrawal are outgrown throughout early childhood and not possible to observe when the children reach 4 years. There are many other factors that will impact development, for instance the verbal atmosphere surrounding the infant such as caregiver-infant conversation [35] which is information this study lack. Another limitation to the study, is that we only have measures of social withdrawal when the children are in infancy, and we cannot track trajectories of social withdrawal later. Notably, the EDEN study also included measures of mental health (SDQ) and found associations with mental health problems when the children were 5–6 years [16, 17]. We did not include measures on socio-emotional development and mental health at 4 years, which could explain the lack of relationship with child outcomes at this age.

Strengths of the current study includes the large sample size and the close follow-up of the children and their families. Other strengths are the thorough assessments of early child development and intellectual functioning at four time points until the children`s fourth birthday. Limitations to the study is the restricted inclusion criteria limiting both the variability of the data and the generalizability of findings. Although using well-accepted and widely used tools to measure neurodevelopment, these are developed for a US setting and there is a lack of validated norms for a South Asian population. The use of US norms in the current study does not bias the observed associations, however caution should be made in comparing scores in the current population to scores of other populations. The psychometric qualities of the m-ADBB has been questioned in this study setting [14], increasing the risk for type II errors. Measures of m-ADBB in these children beyond infancy could have yielded further insights into social withdrawal and child development among these young Nepalese children.

## Conclusion

Being socially withdrawn in infancy is related to a time-limited delay in language development in early childhood, but not to cognitive functioning in later childhood.

### Supplementary Information

**Additional file 1:** **Supplementary Table 1.** Candidate variables for the multivariable regression models to identify relevant confounders between m-ADBB score and neurodevelopment in early childhood. **Supplementary Table 2.** Determinants for total ADBB score in 597 Nepalese infants measured at 6-11 months of age. **Supplementary Table 3.** Wechsler Preschool and Primary Scale of Intelligence, 4th edition total and index scores and NEPSY-II subtest scores in 527 children 4 years of age by being socially withdrawn or not in infancy.

** Additional file 2:** **Supplementary Figure 1.** Bayley-III Expressive and Receptive communication subscales in early childhood in Nepalese children socially withdrawn (*n*=61) or not (*n*=466) in infancy. Legend: Marginal means from linear mixed effects models with the dichotomized m-ADBB score and time included as fixed effects and the individual as random effect and adjusted for age at ADBB measurement, whether family lives in a rented house, low birth weight and whether child is cared for by one of three regular carers when parents are away. In these models we also included an interaction term between time and the ADBB variable. Blue line represents children with no social withdrawal behaviour in infancy, red line children with infant withdrawal in infancy.

## Data Availability

Data available on request. To meet ethical requirements for the use of confidential patient data, requests must be approved by the Nepal Health Research Council (NHRC) and the Regional Committee for Medical and Health Research Ethics in Norway. Requests for data should be sent to the authors, by contacting Child Health Research Project, Department of Child Health, Institute of Medicine, Tribhuvan University (chrp2015@gmail.com), or by contacting the Department of Global Health and Primary Care at the University of Bergen (post@igs.uib.no).
