# Supplementary material for: Social withdrawal behaviour in Nepalese infants and the relationship with future neurodevelopment; a longitudinal cohort study
Source: BMC Pediatr. 2024 Mar 18;24:195. doi: 10.1186/s12887-024-04658-6 (PMC10946118; doi:10.1186/s12887-024-04658-6)
Supplement: Supplementary file 1 — Additional file 1: Supplementary Table 1. Candidate variables for the multivariable regression models to identify relevant confounders between m-ADBB score and neurodevelopment in early childhood. Supplementary Table 2. Determinants for total ADBB score in 597 Nepalese infants measured at 6-11 months of age. Supplementary Table 3. Wechsler Preschool and Primary Scale of Intelligence, 4th edition total and index scores and NEPSY-II subtest scores in 527 children 4 years of age by being socially withdrawn or not in infancy. [file 12887_2024_4658_MOESM1_ESM.pdf]

Supplementary table 1. Candidate variables for the multivariable regression models to identify relevant confounders between m-ADBB score and neurodevelopment in early childhood.

**Background and socioeconomic status**		
Maternal literacy	categorical	Illiterate; primary, secondary, SLC/intermediate; bachelor; master or above
Paternal literacy	categorical	Illiterate; primary, secondary, SLC/intermediate; bachelor; master or above
Maternal occupation	categorical	No work; agriculture, carpet worker; daily wage earner
Paternal occupation	categorical	No work; agriculture, carpet worker; daily wage earner
Maternal age	continuous	years
Paternal age	continuous	years
Joint or nuclear family	dichotomous	joint/nuclear
House ownership	dichotomous	own/rent
Number of rooms in the home	continuous	number
WAMI* score	continuous	score 0-1
**Child Characteristics**		
Age of the infants in months	continuous	months
Sex of the infants	dichotomous	male/female
Preterm	dichotomous	preterm/term
Low birth weigh	dichotomous	≤ 2500 g
Height for age z-score	continuous	z-score
Weight for age z-score	continuous	z-score
Hospitalization the first month of life	dichotomous	yes/no
Haemoglobin (g/L)**	continuous	g/L
**The HOME environment*** **		
Caregiver spontaneously vocalizes to the child at least twice during the visit	dichotomous	yes/no
Caregiver responds to child`s vocalization with a verbal response	dichotomous	yes/no

Caregiver reports no instances of physical punishment during the past week	dichotomous	yes/no
Caregiver does not scold or criticize the child during the visit	dichotomous	yes/no
Caregiver tends to keep child within visual range and looks at the child quite often	dichotomous	yes/no
Caregiver consciously encourages developmental advances	dichotomous	yes/no
Caregiver structures the child`s day	dichotomous	yes/no
Caregiver believes the child`s behaviour can be changed or modified and is influenced by the parent`s behaviour	dichotomous	yes/no
When the primary caregiver is away, care is provided by one of three regular substitutes	dichotomous	yes/no
Child is not cared for by another child (under 12 years of age)	dichotomous	yes/no
There are some toys, tins, balls, dolls, slates, or material in the house that are appropriate play materials for the child	dichotomous	yes/no
The child has a riding toy or some toy that provides gross motor stimulation	dichotomous	yes/no
Caregiver provides toys or interesting activities for the child during the visit	dichotomous	yes/no
There are some magazines, newspapers, or books visible in the house	dichotomous	yes/no
The caregiver tells the child stories or nursery rhymes at least once a week	dichotomous	yes/no
The caregiver sings to the child everyday	dichotomous	yes/no

* We used a composite WAMI-index to indicate socio-economic status, using variables for water and sanitation access, household wealth (assets), maternal education, and income (36, 37). The WAMI score range from 0 to1 with higher scores indicating higher socio-economic status.

**Hemoglobin concentration was measured in all children by the HemoCue analyzer 201 (Hemocue, Angelholm, Sweden). The HemoCue analyzer was calibrated and maintained as per manufacture's guidelines.

*** To evaluate the children`s home environment, we used a selection of items from the HOME Observation of the Measurement of the Environment (HOME inventory) (38). The HOME inventory is a structured interview and observational tool to measure the psychosocial environment and quality of maternal responsiveness and parental practice. The tool has been used widely in studies on early child development in low-income countries. We selected 16 items from a Bangladeshi adapted version of the Home Inventory that have been found to be a feasible tool in the same population in Nepal (39).

Supplementary table 2. Determinants for total ADBB score in 597 Nepalese infants measured at 6-11 months of age

	Ratio of mean* (95%CI) univariate models	Ratio of mean (95% CI) multivariable model
Age at ADBB measurement	0.88 (0.82, 0.93)	0.89 (0.84, 0.95)
Family lives in a rented house	1.24 (1.01, 1.53)	1.23 (1.0, 1.52)
Born with low birth weight	1.52 (1.21, 1.91)	1.39 (1.09, 1.77)
Hemoglobin (g/L)	0.88 (0.79, 0.98)	0.93 (0.83, 1.05)
Caregiver spontaneously vocalizes to the child at least twice during the visit	0.64 (0.41, 0.99)	0.73 (0.46, 1.15)
Caregiver believes the child`s behavior can be changed or modified and is influenced by the parent`s behavior	0.63 (0.45, 0.88)	0.79 (0.56, 1.12)
When the primary caregiver is away, care is provided by one of three regular substitutes	0.61 (0.40, 0.92)	0.61 (0.40, 0.93)
The child has a riding toy or some toy that provides gross motor stimulation	0.72 (0.58, 0.89)	0.81 (0.65, 1.01)
Caregiver provides toys or interesting activities for the child during the visit	0.82 (0.60, 1.01)	0.89 (0.72, 1.09)

* ratios of mean from poisson regression, a ratio larger than 1 means that the total m-ADBB score increases with the determinant variable and a ratio less than 1 means the score decreases

Supplementary table 3. Wechsler Preschool and Primary Scale of Intelligence, 4th edition total and index scores and NEPSY-II subtest scores in 527 children 4 years of age by being socially withdrawn or not in infancy.

**WPPSI-IV, 42-47 months**	**Infants with social withdrawal behaviour n=61**	**Infants without social withdrawal behaviour n=466**	**mean diff (95%CI), p-value* crude**	**mean diff (95%CI), p-value* adjusted****
Full scale	84.3 (8.3)	84.8 (8.6)	-0.5 (-2.8, 1.8	0.5 (-2.2, 2.3)
Verbal comprehension index	82.5 (6.5)	84.2 (8.0)	-1.7 (-3.8, 0.4)	-1.4 (-3.5, 0.7)
Receptive vocabulary subtest	6.5 (1.4)	6.9 (1.8)	-0.5 (-0.9, 0.0)	-0.4 (-0.9, 0.1)
Information subtest	6.9 (1.9)	7.1 (1.9)	-0.2 (-0.7, 0.3)	-0.8 (-0.6, 0.4)
Visuo-spatial index	86.6 (8.4)	85.5 (8.4)	1.2 (-1.1, 3.4)	1.6 (-0.7, 3.9)
Block design subtest	8.7 (1.9)	8.7 (1.9)	0.0 (-0.5, 0.5)	0.1 (-0.5, 0.6)
Working-memory index	102.0 (14.6)	103.8 (13.1)	-1.8 (-5.4, 0.5)	-0.9 (-4.4, 2.7)
Picture memory subtest	9.0 (2.9)	9.1 (2.6)	-0.1 (-0.9, 0.6)	0.0 (-0.7, 0.7)
Zoo location subtest	11.5 (2.9)	12.1 (2.5)	-0.6 (-1.2, 0.1)	-0.4 (-1.1, 0.3)
