# Supplementary figures and images for: Social withdrawal behaviour in Nepalese infants and the relationship with future neurodevelopment; a longitudinal cohort study
Source: BMC Pediatr. 2024 Mar 18;24:195. doi: 10.1186/s12887-024-04658-6 (PMC10946118; doi:10.1186/s12887-024-04658-6)

Expressive communication

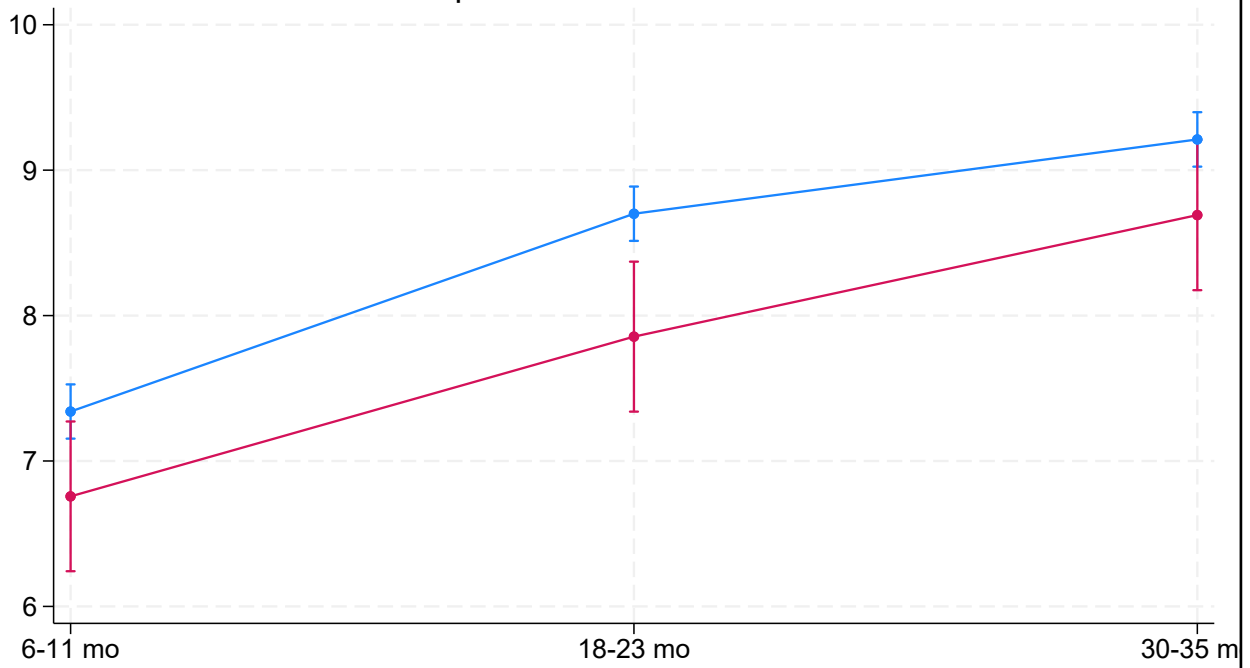

Receptive communication

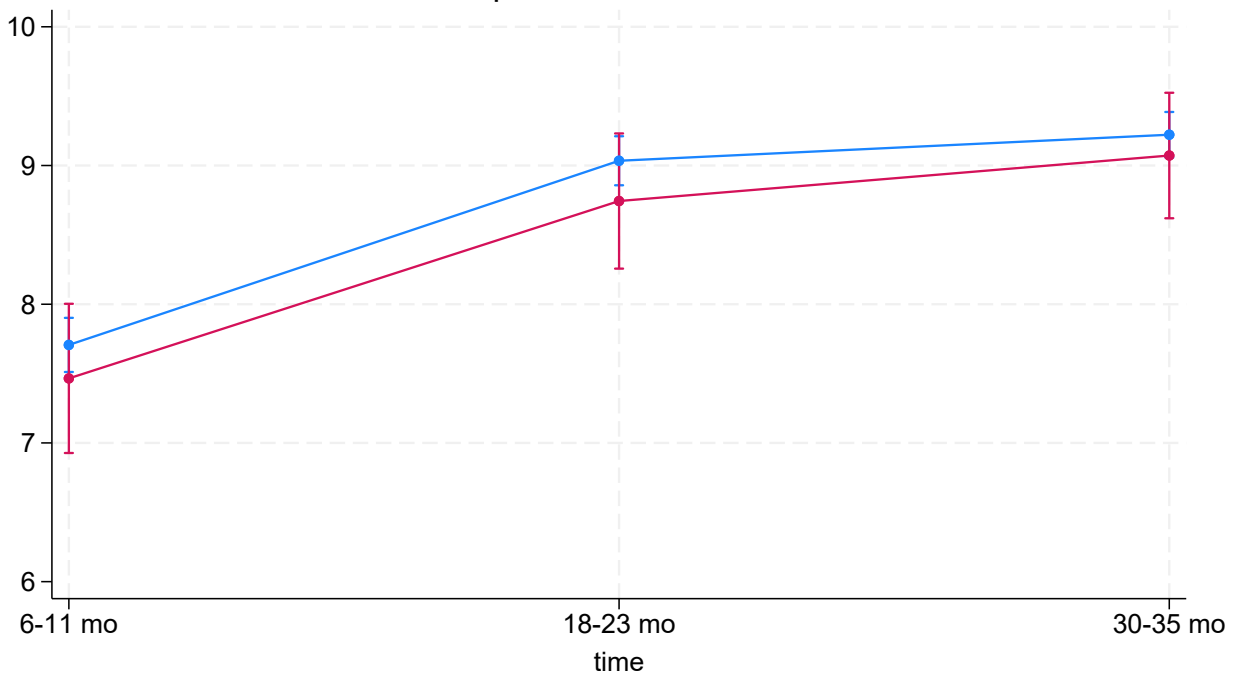

Supplement: Supplementary file 2 — Additional file 2: Supplementary Figure 1. Bayley-III Expressive and Receptive communication subscales in early childhood in Nepalese children socially withdrawn (n=61) or not (n=466) in infancy. Legend: Marginal means from linear mixed effects models with the dichotomized m-ADBB score and time included as fixed effects and the individual as random effect and adjusted for age at ADBB measurement, whether family lives in a rented house, low birth weight and whether child is cared for by one of three regular carers when parents are away. In these models we also included an interaction term between time and the ADBB variable. Blue line represents children with no social withdrawal behaviour in infancy, red line children with infant withdrawal in infancy. [file 12887_2024_4658_MOESM2_ESM.pdf]
